# Testing for genetic trade-offs between early- and late-life reproduction in a wild red deer population

**DOI:** 10.1098/rspb.2007.0986

**Published:** 2008-01-23

**Authors:** Daniel H Nussey, Alastair J Wilson, Alison Morris, Josephine Pemberton, Tim Clutton-Brock, Loeske E.B Kruuk

**Affiliations:** 1 Large Animal Research Group, Department of Zoology, University of CambridgeCambridge CB2 3EJ, UK; 2 Institute of Evolutionary Biology, University of EdinburghEdinburgh EH9 3JT, UK

**Keywords:** ageing, antagonistic pleiotropy, disposable soma, random regression, senescence, trade-off

## Abstract

The antagonistic pleiotropy (AP) theory of ageing predicts genetically based trade-offs between investment in reproduction in early life and survival and performance in later life. Laboratory-based research has shown that such genetic trade-offs exist, but little is currently known about their prevalence in natural populations. We used random regression ‘animal model’ techniques to test the genetic basis of trade-offs between early-life fecundity (ELF) and maternal performance in late life in a wild population of red deer (*Cervus elaphus*) on the Isle of Rum, Scotland. Significant genetic variation for both ageing rates in a key maternal performance measure (offspring birth weight) and ELF was present in this population. We found some evidence for a negative genetic covariance between the rate of ageing in offspring birth weight and ELF, and also for a negative environmental covariance. Our results suggest rare support for the AP theory of ageing from a wild population.

## 1. Introduction

There is now broad theoretical agreement that ageing, or senescence, evolves because extrinsically caused mortality leads to a reduction in the force of natural selection with age (Hamilton 1966; Partridge & Barton 1996). Senescence is thought to evolve as a result of two mutually non-exclusive genetic mechanisms: antagonistic pleiotropy (AP; Williams 1957) and mutation accumulation (Medawar 1946; Hamilton 1966). The predictions of the evolutionary theory of ageing have generally been supported in experimental laboratory studies of model organisms like *Drosophila melanogaster* (Rose 1984; Stearns *et al.* 2000). However, the generality of the evolutionary theory of ageing for populations experiencing natural conditions has recently been the subject of both theoretical and empirical challenge (Rose 1991; Reznick *et al.* 2004; Baudisch 2005; Williams *et al.* 2006). To date, only a handful of studies have examined the genetic architecture underlying the ageing process in wild vertebrate populations (Charmantier *et al.* 2006*a*,*b*).

Under the AP theory, the weakening of natural selection with age results in alleles with beneficial early-life effects but detrimental late-life effects being favoured by selection (Williams 1957). A physiological variant of the AP theory, known as the ‘disposable soma’ hypothesis, views ageing as the accumulation of cellular wear and tear with selection acting on genetic variation in investment in repair of damage to the soma (Kirkwood & Holliday 1979; Kirkwood & Rose 1991). Both theories predict that negative genetic correlations should exist between life-history traits expressed in early versus late life (Williams 1957; Kirkwood & Rose 1991). Experimental laboratory-based work has demonstrated negative genetic correlations between early reproduction and survival in later life (Rose 1984, 1991). Several recent studies of phenotypic variation in wild vertebrate populations have demonstrated long-term trade-offs between early reproduction and either rates of decline in reproductive performance in old age or survival probabilities in later life (Gustafsson & Part 1990; Reid *et al.* 2003; Nussey *et al.* 2006). However, the genetic basis of such trade-offs in natural populations has rarely been tested (Charmantier *et al.* 2006*b*).

**Figure 1:**
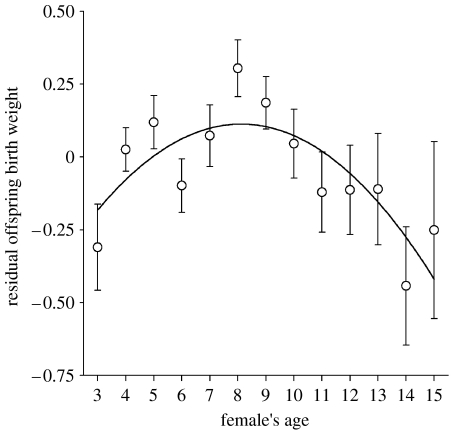
Mean residual offspring birth weight (±s.e. bars) for each female age class with a quadratic regression fitted through points. The residuals are from a linear model of offspring birth weight controlling for offspring sex, date of birth, birth year and the female's reproductive status, age at first reproduction, age at last reproduction and its quadratic.

Here we test for negative genetic correlations between investment in early-life reproduction and maternal ageing rates in a wild population of red deer (*Cervus elaphus*) on the Isle of Rum, Scotland. A key indicator of maternal performance in this population is offspring birth weight (Clutton-Brock *et al.* 1982; Coulson *et al.* 2003). This trait has previously been shown to vary as a quadratic function of female's age: offspring birth weights increased from 3 years of age, peaking at approximately 8 years, and then showed an accelerating decline indicative of senescence (Coulson *et al.* 2003; Nussey *et al.* 2006; figure 1). In a previous study of this population conducted at the phenotypic level, we demonstrated that females with high fecundity in early adulthood (less than 9 years old) had faster rates of senescence in offspring birth weight in later life (Nussey *et al.* 2006).

If this trade-off was the product of AP, we would expect genes that increased early-life fecundity (ELF) to be associated with increasingly poor maternal performance with age. A previous study of wild mute swans was able to demonstrate genetic correlations between age at first and last reproduction that supported AP theory of ageing (Charmantier *et al.* 2006*b*). However, as yet no study has examined genetic correlations affecting age-dependent trait expression in the wild. Here we use life-history and pedigree information from the Rum red deer population and quantitative genetic models to test for additive genetic variation in maternal ageing rates and genetic trade-offs between early and late life in this population. Specifically, we use multivariate random regression animal models to quantify age-related changes in genetic variance for maternal reproduction performance, and its genetic covariance with ELF.

## 2. Material and methods

### (a) Study population and pedigree

Red deer in the North Block of the Isle of Rum, Scotland, have been the subject of an individual-based study since 1971. Individual deer are recognizable as a result of artificial markings and natural idiosyncrasies. Regular censusing of the population and mortality searching during winter mean that complete life-history data are available for most deer resident to the North Block (Clutton-Brock *et al.* 1982). Females can begin breeding at 3 years of age, giving birth to a maximum of a single calf per year. Close observation of females and their newborn offspring during the calving season (late May–early July) enables the capture of approximately 80% of calves born in the study area within a few days of birth. Calves are weighed, sexed and artificially marked at capture. Offspring birth weight was calculated using data on calf weight at capture and estimated time of birth (following Clutton-Brock *et al.* 1982). ELF was calculated as the number of offspring produced by a female up to and including 8 years of age. We used 1667 birth weight observations from offspring of 408 red deer females born in the North Block population between 1971 and 2006 in our analyses.

The population pedigree was reconstructed using a combination of behavioural and genetic methods. Regular observation and individual identification allowed mother–offspring relationships to be determined with certainty. Since 1982, tissue and blood samples have been taken from calves at capture for genotyping. Additionally, over 300 animals born prior to this date were sampled by chemical immobilization or post-mortem. Paternities were assigned genetically, using up to 15 highly variable microsatellite loci, with the program Cervus (Marshall *et al.* 1998). We included all males observed holding harems during a rut as potential candidate fathers for calves born in the subsequent spring. Genetic paternities were assigned where the confidence score given by Cervus was more than or equal to 80%, and there was no more than one parent–offspring mismatch. Behavioural methods were used where no genetic paternity was assigned. Males were assigned paternities if they held the calf's mother in their harem for more than 5 days during the 11-day window around estimated conception (235 days backdated from the date of parturition; see Clutton-Brock *et al.* 1997). This criterion corresponds extremely well with genetic assignment techniques (J. Pemberton 2007, unpublished data). Since 1971, of the 3122 deer born in the study area, 1061 were assigned paternities using genetic methods and an additional 291 by behavioural methods.

### (b) Quantitative genetic analysis

We used random regression ‘animal model’ techniques (Kruuk 2004; Wilson *et al.* 2005) to: (i) partition variance in ELF and ageing rates in offspring birth weight into additive genetic and environmental components and (ii) estimate the additive genetic and environmental covariances across ages in these two traits. Throughout, we considered offspring birth weight as a trait of the mother (following Nussey *et al.* 2006). To avoid potential bias arising from small sample sizes for the very oldest age classes, we considered only females aged 15 or younger. The population average ageing rate for offspring birth weight is well described by a quadratic function of female's age, once other factors influencing the trait have been accounted for (Coulson *et al.* 2003; Nussey *et al.* 2006; figure 1). Female's age and its quadratic were therefore fitted as fixed covariates in all models of offspring birth weight. In all models, age and its quadratic were mean-centred so that model intercepts reflected offspring birth weights in prime age. All models were run in ASReml v. 2.00a (VSN International).

We initially modelled offspring birth weight (*y*)of female (*i*) at occasion (*j*) as

$$y_{ij}=\mu +\beta_{1}{\text{age}}_{j}+\beta_{2}{\text{age}}_{j}^{2}+(\text{other}\;\text{fixed}\;\text{effects})+a_{i}+{\text{pe}}_{i}+{\text{year}}_{j}+e_{i},$$

where *μ* is the population mean and *β*_1_ and *β*_2_ are the population mean age slope and its quadratic, respectively. Other fixed-effect terms included were offspring sex, female's reproduction status, offspring birth date, female's age at first reproduction and female's age at last reproduction and its quadratic (following Nussey *et al.* 2006). *a*_*i*_ is the additive genetic effect, estimated as the covariation between relatives weighted by coefficients of co-ancestry generated from the population pedigree (Kruuk 2004), pe_*i*_ is the permanent environment effect on individual *i* (Kruuk & Hadfield 2007), year_*j*_ is the effect of breeding year and *e*_*i*_ is the residual environmental effect. These random effects are assumed to have a mean of zero and variances equal to $\sigma_{\text{A}}^{2}$, $\sigma_{\text{PE}}^{2}$, $\sigma_{\text{YEAR}}^{2}$ and $\sigma_{\text{R}}^{2}$, respectively. Throughout our analyses, we assumed $\sigma_{\text{R}}^{2}$ to be constant with respect to age.

Random regression models estimate between-group variance in a function (or series of functions) relating a focal trait to a covariate (Henderson 1982). These models have a history of application within both evolutionary biology (Kirkpatrick & Heckman 1989; Gomulkiewicz & Kirkpatrick 1992) and animal breeding (see Schaeffer (2004) for a review). Recently, they have been used to model phenotypic plasticity and ageing rates in wild vertebrate populations (Nussey *et al.* 2005; Wilson *et al.* 2005). Random regression models estimate the variance and the covariance in functions describing the change in a trait of interest over a covariate, such as age or environment (Schaeffer 2004; Wilson *et al.* 2005). In the simplest case, a linear function is used and individual phenotype is expressed as two terms: an intercept (equivalent to trait value at the mean environment or age) and a slope (the change in trait with age or environment). A simple random regression model for a trait using age as a covariate would therefore estimate variance between individuals in trait elevation, variance in the linear slope of the trait on age and the covariance between elevation and slope. (Co)variance components of more complex relationships can be modelled by incorporating higher-order polynomial functions (Wilson *et al.* 2005). The covariance functions estimated in random regressions can be used to derive age-specific predictions of variance components in the focal trait (as in Wilson *et al.* 2005, 2006).

Between-individual phenotypic variance in covariance functions estimated in a random regression can be further decomposed within an animal model framework into additive genetic and permanent environmental components (Schaeffer 2004; Wilson *et al.* 2005; Nussey *et al.* 2007). We extended the animal model of offspring birth weight described above to examine variation in individual's deviation from the population mean ageing curve (figure 1), using a random regression approach, as follows:

$$y_{ij}=\mu +\beta_{1}{\text{age}}_{j}+\beta_{2}{\text{age}}_{j}^{2}+(\text{other}\;\text{fixed}\;\text{effects})+a_{i}+a_{\text{age}\;i}{\text{age}}_{j}+{\text{pe}}_{i}+{\text{pe}}_{\text{age}\;i}{\text{age}}_{j}+{\text{year}}_{j}+e_{i},$$

where *a*_*i*_ and pe_*i*_ represent the intercept of the additive genetic and permanent environment effect, respectively. The terms *a*_age *i*_age_*j*_ and pe_age *i*_age_*j*_ are the linear slopes with age at additive genetic and permanent environment levels, respectively. This model therefore estimates the variance in intercept and slope and their covariance as random effects at both additive genetic and permanent environment levels. By including the fixed effects for age and its quadratic (*β*_1_ and *β*_2_, respectively), the variances in elevation and slope are estimated as deviations from this population mean ageing curve. We tested the significance of the slope terms using model comparison based on likelihood-ratio tests (LRTs). We ran models including additional additive genetic and permanent environment quadratic functions with age, but these did not significantly improve the model fit (additive genetic effect added: *Χ*_3_^2^=6.25, *p*=0.10) or caused the model to fail to converge (permanent environment effect).

In addition, we ran an animal model of ELF with only additive genetic and residual environmental effects and no fixed-effect terms. ELF is measured once across an individual's lifetime and so permanent environment and annual effects cannot be measured. Maternal and birth cohort effects on ELF were tested by fitting mother's identity and year of birth as random effects, but LRTs revealed that these were not significant. We tested the significance of the additive genetic variance component for ELF by comparing models with and without this effect fitted using LRTs.

We found evidence for significant additive genetic variation for both ELF and ageing rates in offspring birth weight (see §3). Therefore, we ran a bivariate random regression animal model (including all fixed effects for each of the two traits as described above) of ELF and offspring birth weight. Since ELF did not vary with age, only variance in its intercept could be estimated, while variance in both elevation and slope with age could be estimated for offspring birth weight. The model produced a 3×3 additive genetic (co)variance matrix between ELF elevation, offspring birth weight elevation, and slope of offspring birth weight with age. The residual environmental covariance between offspring birth weight and ELF was also estimated. The significance of covariance terms was determined by fixing their values to zero in turn and comparing models using LRTs. The additive genetic covariance function matrix for ELF and offspring birth weight was back-transformed to give ‘G’, a matrix containing predicted age-specific additive genetic variance for both traits and the additive genetic covariance between them. Approximate standard errors were generated for these (co)variances following Fischer *et al.* (2004). To check that the results obtained in the above analysis were not driven by genetic or environmental effects specific to females that died in early adulthood, we re-ran the above bivariate animal models of offspring birth weight including only data from female deer aged 9 years or more.

## 3. Results

**Figure 2:**
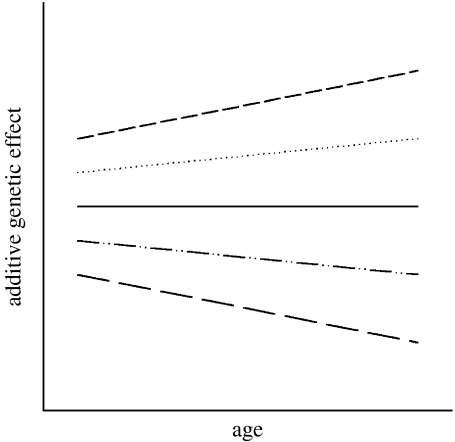
The pattern of variation in genotypic reaction norms across ages for offspring birth weight (relative to the population mean age curve in figure 1) implied by the genetic (co)variance structure in our final model (table 1). The lines on the plot illustrate the change in an individual's breeding value with age. The plot is purely illustrative of the broad pattern and is not based on any quantitative estimates from the model.

We found significant additive genetic variance for both female ELF and ageing rates in offspring birth weights. Inclusion of an additive genetic effect in the univariate models of ELF significantly improved the model fit (*Χ*_1_^2^=6.00; *p*=0.015). In the univariate models of offspring birth weight, the additive genetic term for individual slope was significant (*Χ*_2_^2^=21.56; *p*<0.001), as was covariance between genetic elevation and slope terms for offspring birth weight (*Χ*_1_^2^=7.708; *p*=0.006). The permanent environment slope term was not significant (*Χ*_2_^2^=1.37; *p*=0.50) and was dropped from all subsequent models. The genetic covariance between elevation and slope was positive, suggesting an outward fanning pattern of genetic reaction norms (see figure 2 for illustration), and implying that $\sigma_{\text{A}}^{2}$ for offspring birth weight must be increasing with age.

In the bivariate random regression animal model, additive genetic and residual environmental components of variation in offspring birth weight were negatively correlated with ELF. The genetic and residual environment (co)variances estimated in the bivariate model are presented in table 1*a*. Residual covariance between the traits was significant and negative (*Χ*_1_^2^=6.56; *p*=0.01; table 1*a*). There was also a marginally non-significant, negative additive genetic covariance between ELF and the slope term for offspring birth weight (*Χ*_1_^2^=3.54; *p*=0.07; table 1*a*). The genetic covariance between ELF and the intercept of offspring birth weight was weak and non-significant (*Χ*_1_^2^=0.18; *p*=0.67; table 1*a*). The negative genetic correlation between offspring birth weight slope and ELF (*r*_A_=−0.54) reveals that genes for increased ELF are associated with more rapid declines in offspring birth weight with age. The absence of genetic covariation between offspring birth weight and ELF implies that genes affecting ELF did not influence offspring birth weights in prime age.

Re-running the above bivariate random regression model having excluded offspring birth weights from females in early adulthood (aged below 9 years), yielded qualitatively similar results (table 1*b*). In all the cases, the estimated variance and covariance terms were in the same direction and of similar magnitude, but had larger standard errors due to the restricted sample size (641 observations from 247 females rather than 1667 observations from 408 females). Additive genetic variation for ageing rates in reproductive performance remained significant among these older females (slope term for offspring birth weight: *Χ*_2_^2^=6.15; *p*=0.046; table 1*b*). The genetic correlation between ELF and the age slope for offspring birth weight remained negative and was almost identical in magnitude to correlation in the full model (*r*_A_=−0.55) but, presumably owing to the substantially reduced sample size and therefore power in the restricted model, no longer approached significance (*Χ*_1_^2^=1.15; *p*=0.28; table 1*b*). The consistency of direction and magnitude of the estimates in table 1*a*,*b* suggests that the patterns observed here were not due to effects specific to early adulthood.

**Figure 3:**
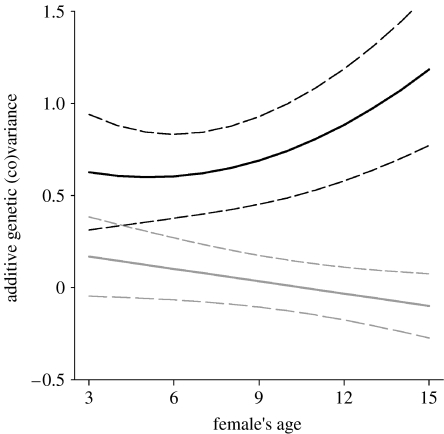
The estimated change in the additive genetic variance in offspring birth weight (solid black line) and in the genetic covariance between offspring birth weight and early-life fecundity (solid grey line) with female's age. Estimated 95% CIs (dashed lines) are plotted. Additive genetic variance for early-life fecundity was 0.289 and constant with age (table 1*a*).

The additive genetic covariance functions in table 1*a* were back-transformed to generate a *G* matrix of age-specific predictions of additive genetic variance for ELF and offspring birth weight and of covariance between the traits. Age-specific predictions of additive genetic variance in offspring birth weight increased with age (figure 3). Although variance components for ELF were by definition constant with age (the trait was measured once across an individual's lifetime), its genetic covariance with offspring birth weight could vary with age. The back-transformed *G* matrix revealed that the additive genetic covariance between ELF and offspring birth weight declined with age (figure 3). The estimated genetic correlation (*r*_A_) between these two traits changed from +0.40 at age 3 to −0.17 at age 15.

## 4. Discussion

The results of our univariate random regression model of offspring birth weight clearly demonstrate that additive genetic variation for maternal ageing rates is present in this population. Genetic variance for ageing is an implicit assumption of the evolutionary theory of ageing, but this assumption has rarely been tested outside of laboratory settings (Rose 1991). Interestingly, a pattern of increasing $\sigma_{\text{A}}^{2}$ with age similar to that observed here for offspring birth weight (figure 3) has also been observed in a wild population of mute swans (*Cygnus olor*) in a fitness-correlated trait (Charmantier *et al.* 2006*a*). An increase in $\sigma_{\text{A}}^{2}$ with age in fitness-correlated traits is consistent with a decrease in the force of natural selection with age (Charlesworth 2001), and is potentially predicted by both AP and mutation accumulation mechanisms for the evolution of senescence (Charlesworth & Hughes 1996).

The results from our bivariate random regression animal models suggest that the previously documented trade-offs between ELF and maternal ageing rates in this population may have some genetic basis. The increasingly negative genetic correlation between ELF and offspring birth weight with age (figure 3) could be indicative of the presence of allelic variation at loci with antagonistic effects on early- versus late-life reproduction or condition (Williams 1957). It could also be consistent with the presence of a genetic variation for investment in growth/reproduction versus somatic maintenance (Kirkwood & Holliday 1979). The significantly positive genetic correlation between elevations and slopes observed here implies an outward fanning pattern of genetic reaction norms across age, with some individuals performing increasingly poorly relative to the population mean with age, while others perform increasingly well relative to the population mean (illustrated in figure 2). This pattern may be indicative of genetic variation in the capacity for continued somatic maintenance across lifespan. If so, the negative genetic covariance between the offspring birth weight slope term and ELF (table 1) suggests that genes for continued somatic maintenance are associated with reduced early reproduction.

Both explanations given above are consistent with life-history theories of ageing and, specifically, some form of AP underlying the evolution of ageing (Williams 1957; Partridge & Barton 1996). The negative genetic covariance observed here between maternal ageing rates and early-life reproduction in wild deer would be expected to constrain selection against the deleterious effects of ageing, and could maintain additive genetic variation in ageing rates. The bivariate models also revealed that non-genetic, residual environmental components of variation in ELF and offspring birth weight were negatively correlated in this population (table 1*a*), suggesting that both environmental and genetic factors could underlie the observed trade-off at the phenotypic level. However, our analyses do assume that residual environmental variance in offspring birth weight is constant with age and so this finding should be interpreted with caution. Models estimating age-specific residual variance components have been applied to univariate analyses in wild pedigreed populations (Wilson *et al.* 2005), but it is unclear how this could be achieved within a bivariate animal model in which one trait has multiple records across lifespan but the other does not. Estimating the relative influence of environmental and genetic factors on early- versus late-life trade-offs is likely to prove challenging using data from pedigreed natural populations, but represents an important avenue for future research.

The present study represents the first application of a bivariate random regression to model age-specific patterns of genetic covariance between traits in a wild vertebrate population. Our analyses illustrate the potential importance of such techniques for understanding the genetic architecture that underpins ageing in nature. The use of random regression allows the estimation of age-specific (co)variance components without the loss of power suffered by splitting data into separate age classes (Wilson *et al.* 2005; Nussey *et al.* 2007). Standard errors on genetic correlations are typically high when pedigreed wild populations are studied, and therefore maximizing power to detect changes in genetic or environmental covariance across age or environment may be particularly important in such analyses. Although we observed a negative genetic correlation in the direction predicted by the AP and disposable soma theories of ageing, this effect was marginally non-significant. It is worth noting that the Rum red deer dataset comprises one of the most complete pedigrees and longitudinal life-history records of any wild vertebrate study system, and so statistical power to detect effects of this kind using bivariate models is likely to be an issue in most studies of wild pedigreed populations. However, we believe that further development and application of this approach will yield important insight into the genetic and environmental factors influencing the life-history constraints underlying patterns of ageing in natural populations.

## Figures and Tables

**Table 1:** Variance and covariance components (with standard errors in brackets) from a bivariate ‘animal model’ of female early-life fecundity (ELF) and offspring birth weight (BW). The model included residual (co)variance terms for both traits, and additive genetic (co)variance terms for the intercept and slope of BW with age and the intercept term for ELF. Permanent environment and year terms were included for BW only. Fixed effects were fitted to BW only and are described in §2. (*a*) Components from the model including all data (1667 offspring birth weights from 408 females). (*b*) An analysis of data only from females aged 9 years old or above (641 offspring birth weights from 247 females).

	(*a*) full dataset		(*b*) data from females aged ≥9 years			
		
	BW	ELF		BW	ELF	
*residual*						
BW	0.700 (0.030)			0.757 (0.063)		
ELF	−0.208 (0.072)	1.161 (0.147)		−0.068 (0.087)	0.805 (0.137)	

	BW_intercept_	BW_slope_	ELF	BW_intercept_	BW_slope_	ELF
*additive genetic*						
BW_intercept_	0.690 (0.119)			0.827 (0.213)		
BW_slope_	0.140 (0.057)	0.215 (0.070)		0.289 (0.101)	0.179 (0.100)	
ELF	0.035 (0.073)	−0.134 (0.069)	0.289 (0.142)	0.011 (0.085)	−0.110 (0.076)	0.225 (0.135)
*permanent environmental (offspring birth weight only)*						
BW	0.026 (0.067)			0.112 (0.128)		
*year (offspring birth weight only)*						
BW	0.095 (0.029)			0.107 (0.044)		
